# Actual perspectives on LDLT in Europe

**DOI:** 10.1007/s13304-024-01933-0

**Published:** 2024-07-04

**Authors:** Stefanie Josefine Hehl, Henrik Petrowsky, Silvio Nadalin

**Affiliations:** 1 Department of General, Visceral and Transplant Surgery, University Hospital Tübingen, Hoppe-Seyler Strasse 3, 72076 Tübingen, Germany; 2 Department of Surgery, Swiss Hepato-Pancreato-Biliary and Transplantation Centre, University Hospital Zurich, Zurich, Switzerland

In the field of liver transplantation, distinct practices set apart the East and the West (Fig. 1). In the East, cultural and religious beliefs, coupled with advanced surgical expertise, have fostered a robust environment for living donor liver transplantation (LDLT). Conversely, Western Europe predominantly relies on deceased organ donations, grappling with chronic shortages and the challenges of utilizing marginal organs, i.e., extended criteria donor (ECD) and donation after circulatory death (DCD) organs, likely associated with with poorer outcomes and higher rates of post-transplant complications [1]. Yet, the implementation of machine perfusion techniques offers exciting possibilities to improve preservation of organ function, reduce ischemia–reperfusion injury, and assess organ viability in real time. Several working groups have demonstrated that hypothermic oxygenated machine perfusion (HOPE), normothermic machine perfusion (NMP), and sequential perfusion strategies allow for an increased utilization of ECD and DCD liver grafts [2, 3].

**Fig. 1:**
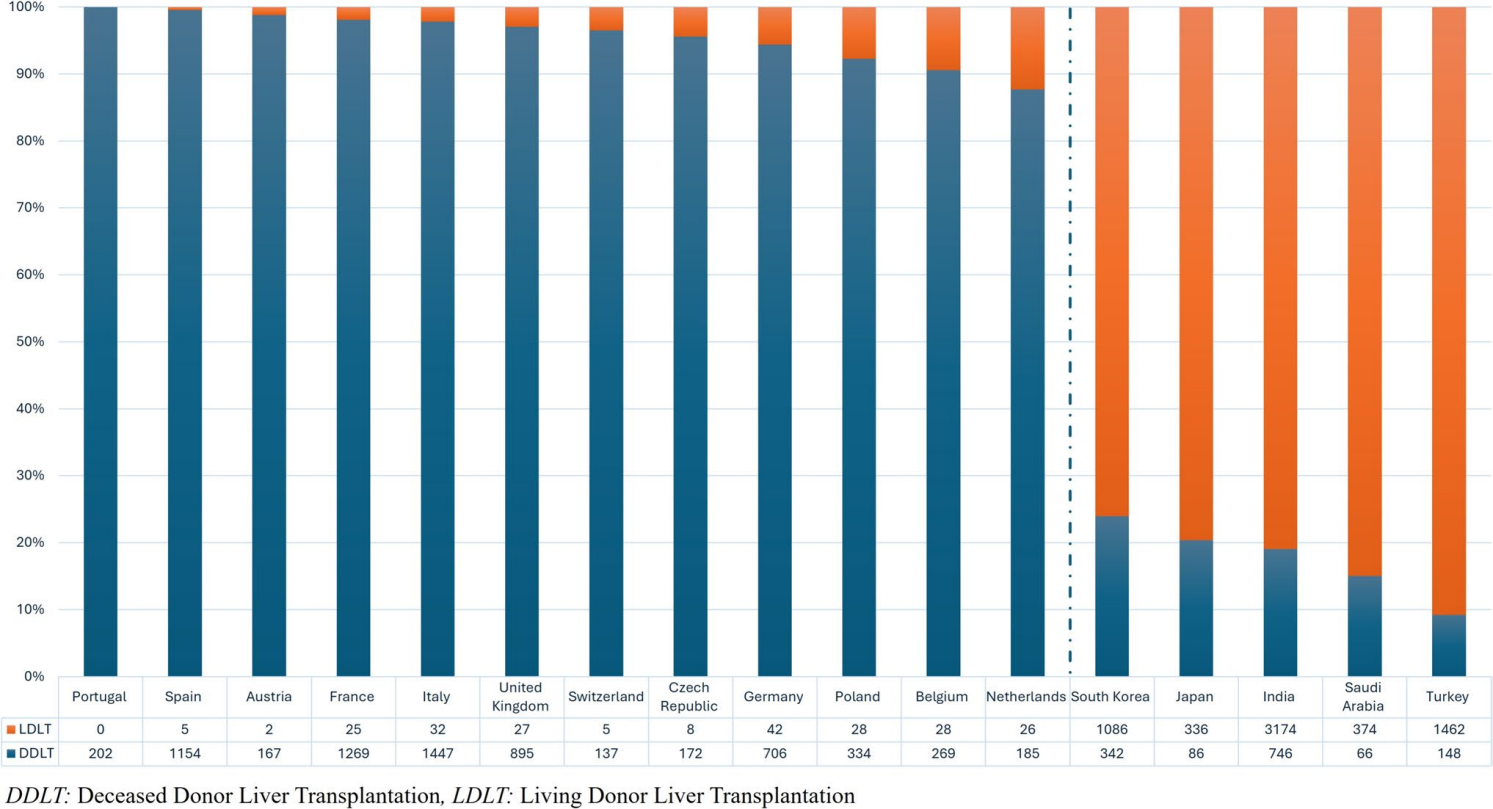
The two worlds of liver transplantation—Western Europe versus Asia in 2002 (absolute numbers and percentage). *DDLT* deceased donor liver transplantation; *LDLT* living donor liver transplantation

Amid these various approaches and their respective challenges, what lies at hand is that the greatest potential resides in LDLT. Utilizing living donation offers the opportunity to overcome the limitations of deceased donor liver transplantation (DDLT) and provides timely access to transplantation for patients in need. Despite this, LDLT still plays a minor role in Europe. While only very few nations have witnessed an increase in LDLT activity, others have experienced stagnation or decrease in procedure numbers over the past 20 years. Turkey is the leading example, being the most active European country in performing LDLT in 2022, displaying an impressive LDLT rate of 90.8% out of a total of 1610 liver transplantations performed. However, there is a substantial disparity when compared to LDLT rates of Western European countries. For instance, the Netherlands reported an LDLT rate of 12.3%, Belgium 9.4% and Germany 5.6% in 2022 (Fig. 1).

When reporting of liver transplantation in Western Europe, we must distinguish between different organizations. Eurotransplant (ET), an international non-profit organization, oversees the organ allocation across eight nations: Austria, Belgium, Croatia, Germany, Hungary, Luxembourg, the Netherlands, and Slovenia, altogether covering a population of approximately 139 million people. Another collaborative organization, the South Alliance for Transplantation (SAT) includes the member countries Spain, France, Italy, Portugal, Switzerland, and the Czech Republic. While SAT member countries adhere to their national allocation rules, they engage in cooperation at various levels within the alliance. Scandiatransplant manages the organ allocation of six Northern European countries comprising Denmark, Finland, Iceland, Norway, Sweden, and Estonia. Both the UK and Poland operate their own organ allocation systems managed by national organizations.

Among ET members, a significant difference exists regarding deceased donor (DD) rates. Austria, Belgium, Croatia, and Slovenia emerge as leading nations with DD rates well above 20 DDs per million people (pmp), while Germany ranks the lowest with only 10.1 DDs pmp. In 2022, the median age of DDs utilized for liver transplantation across all ET countries was 56 years, showing a consistent rise over the past two decades from a median age of 46 in 2004, partially explaining the rise in marginal organs. Among the 1599 liver transplantations performed in the ET area in 2022, 1501 were organs from deceased donors, while only 98 were from living donors, indicating a low LDLT rate of just 6.1% in all of the ET region. 67% of the recipients were children below the age of 16 years with 81% of recipients waiting less than 5 months. Germany, Belgium, and the Netherlands were the top-performing ET members for LDLT in 2022, accounting for 43% (42/98) in Germany, 28.6% (28/98) in Belgium, and 26.5% (26/98) in the Netherlands. Although there is a significant rise in LDLT rates in the Netherlands (as the only ET country) over the past decade (rise from LDLT rates of 3.3% in 2010 to 12.3% in 2022), the LDLT rates in Germany and Belgium are steadily decreasing [4].

Within the nations outside the ET community, a similar trend can be observed. DD represents the primary source of liver grafts with most nations performing DCD transplantations. Spain achieved a remarkable milestone in 2022 with a DD rate of 48 pmp, marking the highest donation activity globally. However, LDLT rates remained minimal across all nations, ranging from 0% in Portugal to 7.7% in Poland (Spain 0.4%, France 1.9%, Italy 2.2%, United Kingdom 2.9%, Switzerland 3.5%, Czech Republic 4.4%) (Fig. 1). Among the Scandiatransplant members, Norway was the sole country conducting LDLT in 2022 with 1 out of 92 liver transplantations performed [5]. LDLT is poorly represented in both ET and non-ET nations with a predominant focus on pediatric patients.

There are several reasons for the lack of success of LDLT in Western Europe. One of the most significant concerns is the risk to the donor’s health. Additionally, regulatory barriers, lack of awareness and misinformation among the public, economic and financial concerns to the donor (e.g., the loss of income during recovery), cultural aspects, and religious beliefs and preferences for DDLT further reduce the acceptance of LDLT within the population. These factors collectively deter both potential donors and recipients, contributing to the limited utilization of LDLT in Western Europe. Comprehensive public education campaigns are crucial for dispelling myths and providing accurate information about the procedure's risks and benefits. Establishing support systems offering psychological counselling, medical follow-up, and financial assistance can alleviate the burdens for donors.

Over recent years, considerable progress has been achieved in addressing donor safety concerns. The implementation of minimally invasive surgical techniques has led to reduced donor morbidity. Notably, robotic LDLT was associated with superior outcomes, a significantly lower donor morbidity but also a lower recipient morbidity, when compared to laparoscopic and open approaches [6]. The introduction of auxiliary liver transplantation procedures like RAPID (Resection and Partial Liver segment 2–3-transplantation with delayed total hepatectomy) will aid in further reducing the donor risk by shifting the risk to the recipient. This is achieved by utilizing smaller grafts (i.e., left or left lateral grafts) particularly in the setting of AA-LDLT [7, 8]. Ultimately, innovations in pharmacological enhancement of liver regeneration, such as MKK4 inhibitors, may further support this approach by reducing the necessity for large liver grafts in the future [9].

In conclusion, the European landscape of LDLT is characterized by challenges, aspirations, and future prospects. New methods, such as minimally invasive surgery and LD- RAPID as well as pharmacological innovations, offer promising avenues to decrease the donor risk with potential for higher acceptance of living donation, both among professionals and the public. By embracing innovation, fostering collaboration, and addressing existing challenges, it is time for Western European countries to improve the utilization of LDLT, while continuing the great efforts in DDLT, enhancing patient access to transplantation and ultimately saving more lives.

## Data Availability

Not applicable.
